# Microbiome of Field Grown Hemp Reveals Potential Microbial Interactions With Root and Rhizosphere Soil

**DOI:** 10.3389/fmicb.2021.741597

**Published:** 2021-11-15

**Authors:** Bulbul Ahmed, Lawrence B. Smart, Mohamed Hijri

**Affiliations:** ^1^Institut de Recherche en Biologie Végétale, Université de Montréal, Montréal, QC, Canada; ^2^Horticulture Section, School of Integrative Plant Science, Cornell AgriTech, Cornell University, Geneva, NY, United States; ^3^African Genome Center, Mohammed VI Polytechnic University (UM6P), Ben Guerir, Morocco

**Keywords:** *Cannabis sativa*, rhizosphere, microbiome, bacterial communities, fungal communities, network analysis, microbial ecology

## Abstract

Hemp (*Cannabis sativa* L.) is a crop bred and grown for the production of fiber, grain, and floral extracts that contribute to health and wellness. Hemp plants interact with a myriad of microbiota inhabiting the phyllosphere, endosphere, rhizoplane, and rhizosphere. These microbes offer many ecological services, particularly those of below ground biotopes which are involved in nutrient cycling, uptake, and alleviating biotic and abiotic stress. The microbiota communities of the hemp rhizosphere in the field are not well documented. To discover core microbiota associated with field grown hemp, we cultivated single *C. sativa* cultivar, “TJ’s CBD,” in six different fields in New York and sampled hemp roots and their rhizospheric soil. We used Illumina MiSeq amplicon sequencing targeting 16S ribosomal DNA of bacteria and ITS of fungi to study microbial community structure of hemp roots and rhizospheres. We found that Planctobacteria and Ascomycota dominated the taxonomic composition of hemp associated microbial community. We identified potential core microbiota in each community (bacteria: eight bacterial amplicon sequence variant – ASV, identified as *Gimesia maris, Pirellula sp. Lacipirellula limnantheis, Gemmata sp.* and unclassified Planctobacteria; fungi: three ASVs identified as *Fusarium oxysporum*, *Gibellulopsis piscis*, and *Mortierella minutissima*). We found 14 ASVs as hub taxa [eight bacterial ASVs (BASV) in the root, and four bacterial and two fungal ASVs in the rhizosphere soil], and 10 BASV connected the root and rhizosphere soil microbiota to form an extended microbial communication in hemp. The only hub taxa detected in both the root and rhizosphere soil microbiota was ASV37 (*Caulifigura coniformis*), a bacterial taxon. The core microbiota and Network hub taxa can be studied further for biocontrol activities and functional investigations in the formulation of hemp bioinoculants. This study documented the microbial diversity and community structure of hemp grown in six fields, which could contribute toward the development of bioinoculants for hemp that could be used in organic farming.

## Introduction

Plant microbiota are an important component that can influence essential plant functions positively or negatively. Better understanding of these host-microbe interactions has the potential to offer intervention tools to manipulate microbiota to enhance ecological services (Berg et al., 2020). Root-associated microbes have been shown to improve the systematically induced root exudation of metabolites process and affect root transcriptome and metabolome (Korenblum et al., 2020). Manipulation of plant microbiota thus trigger host biosynthetic and signaling pathways (Berg et al., 2016; Pascale et al., 2019) and brings up opportunities for plant fitness, improved nutrient usage efficiency, and higher crop yields while limiting chemical fertilizers and greenhouse gas emissions in a sustainable manner (Adesemoye et al., 2009; Berg, 2009; Singh and Trivedi, 2017; Ahmed and Hijri, 2021).

*Cannabis sativa* L. is an emerging crop for the production of fiber, grain, and floral extracts that contribute to health and wellness, but is also widely cultivated indoors with a variety of growing substrates, artificial light, and temperature control. Some initial studies have explored ways to maintain yield (Backer et al., 2019; Saloner and Bernstein, 2020; Danziger and Bernstein, 2021) and encourage pathogen-free production (Taghinasab and Jabaji, 2020; Vujanovic et al., 2020), but few scientific studies on plant-associated microbiota have been conducted. Bacterial and fungal communities associated with the root, leaf, flower, and rhizosphere of the hemp cultivar *C. sativa* “Anka,” have recently been studied in six locations in the Finger Lakes region of New York. This study identified a variety of microbes in each compartment, some of which are known to promote plant growth or contribute to plant resistance (Barnett et al., 2020). Barnett et al. (2020) identified candidate core microbiome members for each compartment sampled (Barnett et al., 2020). Recent studies showed that the *C. sativa* rhizosphere microbial community is determined by rhizosphere soil type and cultivar (Winston et al., 2014; Comeau et al., 2020). However, core microbiota provides essential associated microbial functions, as well as linkages between microbiota and their community structures (Zamioudis and Pieterse, 2011; Lebeis, 2014; Delgado-Baquerizo et al., 2018). Plant genotype, on the other hand, has a significant impact on associated microbiota (Sapkota et al., 2015). Hemp microbiota manipulation has been suggested as a potential to help with fiber processing (Law et al., 2020). Thus, significant consideration should be given to the microbiome of field-grown hemp, especially connecting yield and plant health. The development of effective microbiome-based techniques for improving yields and sustainable production of *C. sativa* is currently limited by the lack of understanding of associated microbes in multiple niches shaping the microbiome in relevant field settings.

**FIGURE 1:**
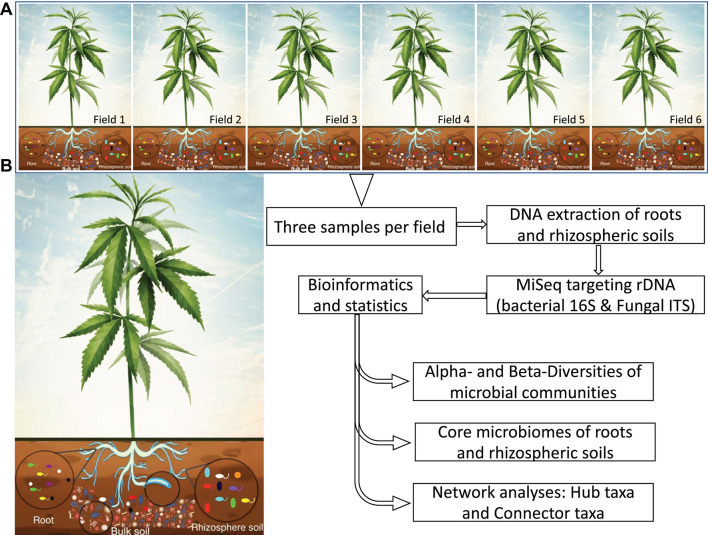
Schematic illustration showing the experimental workflow of a clonal cultivar of hemp grown in six fields distributed at the State of New York **(A)**. MiSeq sequencing and the DADA2 pipeline were used to determine community composition, and core microbes of roots and rhizosphere soils. Network analysis was performed to provide insight into the core microbial taxa and hub microbes. **(B)** Hypothetical core microbes of roots and rhizosphere soils selected from bulk soil microbiome. Rhizosphere soil is show by blue line surrounding roots.

Soil microbiomes provide important ecological services to natural and agricultural ecosystems. Given their potential for positively influencing agricultural productivity, understanding the role of these microbes could enhance our ability to exploit activities that promote efficient soil nutrient use to increase crop yield and quality, while reducing the environmental footprint of agriculture. Given the extraordinary number of microbes that exist in soil and their functional diversity, they could not be examined in depth prior to recent advances in high-throughput sequencing technologies and bioinformatics (Ahmed and Hijri, 2021), that allow to generate a huge amount of sequencing data whose analyses provide an exhaustive taxonomic profile, relative abundance as well as predition of functions. In this study, we used MiSeq amplicon sequencing targeting ribosomal DNA (rDNA) of bacterial 16S and fungal ITS to uncover diversity of roots and rhizosphere soils associated with hemp grown in six different fields located in New York State (United States). We hypothesized that hemp is associated with a core microbiota, and that these taxa have distinct interaction patterns and are diverse in different biotopes. To test our hypothesis, we used high-throughput sequencing technologies to investigate the microbiome of a single clonally propagated cultivar of field grown hemp C. sativa “TJ’s CBD” across six different field locations in New York State, where five plots were conventional and one plot was organic. We determined taxonomic abundance, indicator species, core microbiota, and interkingdom networking for identifying hub microbial taxa, which will help to target, select, and screen microbial taxa with high potential of biostimulant effect on hemp plants (Figure 1).

## Materials and Methods

### Experimental Design and Sampling

The study was performed in six different fields in New York State, United States of America, from May 22 to October 11, 2019 (Table 1), in a randomized complete block design. All plants were started as rooted cuttings treated with Clonex rooting gel in the greenhouse using Lamberts LM-111 potting mix in 50-cell deep flats and transplanted to the field after 25–30 days. Each plot was planted with *C. sativa* “TJ’s CBD,” a clonal cultivar used for cannabidiol production. Only WOF was organic out of six fields. The hemp was planted in black plastic mulch, and weed control was performed by hoeing. Two tons of turkey manure per acre were applied to the WOF plot, and leek (*Allium ampeloprasum* L.) was the prior crop. All rhizosphere soil and root samples were collected in three replicates per field. The root system of each plant was collected using a shovel (approximately 15 cm depth). Fine roots were then cut and put them in a 50 mL tube. Rhizosphere soils were also collected on site and put in a separate container. First, we separated shoots from roots. Roots were gently shacked to remove bulk soil. The remaining soil attached to the roots was collected in Ziploc bags and it was considered as rhizosphere soil (∼500 mg; Floc’h et al., 2020). This rhizosphere soil was visually examined to remove root fragments. Samples were collected in October 2019, and they were immediately transported the lab in an ice pack and stored at −20°C until March 2020. These samples were thawed and used for DNA extractions.

**TABLE 1:** Field locations, planting, and sampling dates.

Field	Altitude	Location	Planting date	Sampling date
Bluegrass Lane (BGL)	42.460647°N, 76.463028W	232 Bluegrass Lane, Ithaca, NY, Tompkins County	June 7, 2019	Oct. 9, 2019
Hudson Valley Lab (HVL)	41.745647°N, 73.967743°W	3357 Rt 9W, Highland NY 12528, Ulster County	June 3, 2019	Oct. 11, 2019
Long Island Horticultural Research and Extension Center (LIH)	40.954766°N, 72.710851°W	3059 Sound Ave., Riverhead, NY 11901, Suffolk County	May 22, 2019	Oct. 1, 2019
McCarthy CBD Trial (MC)	42.895483°N, 77.007547°W	2865 County Rd. 6, Geneva, NY, Ontario County	June 5, 2019	Oct. 10, 2019
McCarthy Stress Trial (MSC)	42.896246°N, 77.008210°W	2865 County Rd. 6, Geneva, NY, Ontario County	July 28, 2019	Oct. 6, 2019
Wegman’s Organic Farm (WOF)	42.776611°N, 77.329667°W	4842 West Lake Rd, Canandaigua, NY Ontario County	July 9, 2019	Oct. 8, 2019

### DNA Extraction, PCR, and Sequencing

DNA extraction from roots and rhizosphere soils and PCR amplifications were carried out as described in Ahmed et al. (2021). Briefly, total 36 frozen hemp root and rhizosphere soil samples were considered for DNA extraction. First, we took 100 mg roots were ground to make fine powder using pre-chilled mortars and pastels. Grounded roots that may contain endophytes were used for DNA extraction using the DNeasy Plant mini kit (Qiagen, Toronto, ON, Canada). DNeasy PowerSoil Pro kit (Qiagen, Toronto, ON, Canada) was used to extract DNA from 250 mg of rhizosphere soil. Eluted DNA (30 μL) was stored at −20°C. The NanoDrop^TM^ 2000/2000c Spectrophotometer (ThermoFisher Scientific, Canada) was used to quantify the extracted DNAs, which were then visualized using a 1 percent agarose gel and the GelDoc System (BioRad, Montreal, QC, Canada). The bacterial 16S rDNA and fungal ITS regions were amplified by PCR. For the amplification of bacterial 16S rDNA, we used forward primer CS1_341 (5′-ACA CTGACGACATGGTTCTACACCTACGGGNGGCWGCAG-3′) and reverse primer CS2_806R (5′-TACGGTAGCAGAGACT TGGTCTGACTACHVGGGTATCTAATCC-3′), while fungal ITS region was amplified with CS1_ITS3_KYO2 (5′-ACACTG ACGACATGGTTCTACAGATGAAGAACGYAGYRAA-3′) and CS2_ITS4 (5′-TACGGTAGCAGAGACTTGGTCTTCCTCCG CTTATTGATATGC-3′; Toju et al., 2012). In addition, 16S bacterial primers were used to target V3 and V4 regions of 16S rDNA while ITS primers targeted ITS2 region located between 5.8S and 25S genes of rRNA. The PCR reactions were performed using 1.5X Platinum^TM^ Direct PCR Universal Master Mix (ThermoFisher, Montreal, QC, Canada), 0.25 μM of each primer, 1.5X Platinum^TM^ GC Enhancer, and 20 ng of template DNA in a 25 μL reaction volume. PCR reactions were run in an Eppendorf Mastercycler Pro S (Eppendorf, ON, Canada) with following cycling conditions: activation at 94°C for 2 min, followed by 35 cycles of denaturation at 94°C for 15 s, annealing at 60°C for 15 s, extension at 68°C for 20 s, a final extension at 68°C for 1 min with a hold at 10°C. Negative PCR controls without DNA were included in each PCR run. PCR products were visualized on 1% agarose gel stained by GelRed on a GelDoc system (BioRad, Saint-Laurent, QC, Canada). Amplicons were sent to the Genome Quebec Innovation Centre for sequencing on an Illumina MiSeq sequencer (San Diego, CA, United States; Montreal, QC, Canada) using 2 X 300 bp pair-end reads which were demultiplexed on the instrument.

### Sequence Processing and Analysis of Data

R4.0.2 (R Core Team, 2020) was used for all bioinformatics tasks, including raw sequencing read processing and graphical analysis. Detailed information on bioinformatics pipeline and data processing are available in the Supplementary Information (Methods 1). Briefly, DADA2 was used to generate the Amplicon Sequence Variants (ASV) table, and taxonomy was assigned to ASV using the reference datasets SILVA (the Silva Project’s version 138) for 16S rDNA (Quast et al., 2013) and UNITE (version 8.3) for ITS (Nilsson et al., 2018). Using R and dplyr v2.0.0 (Wickham and Wickham, 2020), the relative abundance of taxa was calculated. To characterize species diversity in the communities, we used Shannon and Simpson’s index for both abundance and evenness of the species presented in the community. We used the “rrarefy” function of the vegan package v 2.5-6 to normalize the dataset before calculating diversity indices (Oksanen et al., 2020). The Shannon and Simpson diversity indices were calculated using the vegan v 2.5-6 on R. We calculated species evenness using Pielou’s index [*J* = *H*/In (*S*) where *H* is Shannon diversity index and *S* referred the total number of species in the dataset] using vegan package v 2.5-6 on R. Principle Coordination Analysis (PCoA) were calculated using Bray-Curtis distance matrix of Hellinger transformed counts using the R package vegan v 2.5.6. After the calculation of alpha diversity indices by ANOVA, we performed Tukey’s *post-hoc* tests to compare between hemp fields and sample types using package agricolae v1.3-3 (Peşteanu and Bostan, 2020) on R. Permutation-based multivariate analysis of variance (PERMANOVA; Anderson, 2001) was performed on samples considered for investigation of interaction effects of certain drivers on bacterial and fungal community with the function “Adonis” of the R package vegan v 2.5-6 using Hellinger-transformed and permutations 999 (Oksanen et al., 2019). With metacoder v 0.3.4, we visualized taxonomic abundance at the order level (Poisot et al., 2017). According to the definition of “core plant microbiota” (Vandenkoornhuyse et al., 2015), we defined core microbiota which is made up of taxa that are present in 100% of samples, root or rhizosphere soil associated with the host in different fields. We used the package indicspecies v 1.7.9 (De Cáceres and Jansen, 2019) using Šidák correction for multiple comparison in the R package RVAideMemoire v 0.9-78 (Hervé and Hervé, 2020). The algorithm “glasso” of the SPIEC-EASI v 1.0.6 (Kurtz et al., 2015) was used to perform a co-occurrence network analysis and the results were then exported into Cytoscape v 3.8.0 for visualization (Shannon et al., 2003). Edges were described as co-occurrences or mutual exclusions of positive or negative inverse covariance values between nodes. Betweenness centrality and degree emphasize central nodes as well as provide information about network architecture. Betweenness centrality is defined as the ratio of the shortest path between all other nodes in the network involving the given node. A ratio of betweenness centrality and degree of connectivity more than 95% of network taxa may indicate community participation in multipartite co-occurrences, allowing us to define strongly interconnected taxa as hub-taxa. The Venn diagrams were generated using the ‘‘Calculate and draw Venn diagrams’’ tool available on VIB, University of Gent^1^.

## Results

### Sequencing and Bioinformatics

We sampled root and rhizosphere soil of *C. sativa* (hemp) in six fields in New York: Bluegrass Lane (BGL), Hudson Valley Lab (HVL), Long Island Horticultural Research and Extension Center (LIH), McCarthy CBD Trial (MC), McCarthy Stress Trial (MSC), and Wegman’s Organic Farm (WOF). Three plants per field, for a total of 18 plants, were sampled for root and rhizosphere soil. Illumina MiSeq produced a total of 11,617,362 pair-end raw reads (5,639,337 from bacteria and 5,978,025 from fungi). The number of reads per sample ranged from 41,561 to 98,764 for bacteria, and 34,891 to 106,235 for fungi. Using the DADA2 pipeline, 7160 bacterial and 3993 fungal ASVs were obtained. We also removed 26 ASVs from bacteria and 862 ASVs from fungi whose taxonomy belonged to mitochondria or chloroplast, leaving 7134 ASVs for bacteria and 3131 ASVs for fungi, respectively.

### Microbial Diversity Patterns in Field Grown Hemp: Compartment and Field-Dependent Effects

**FIGURE 2:**
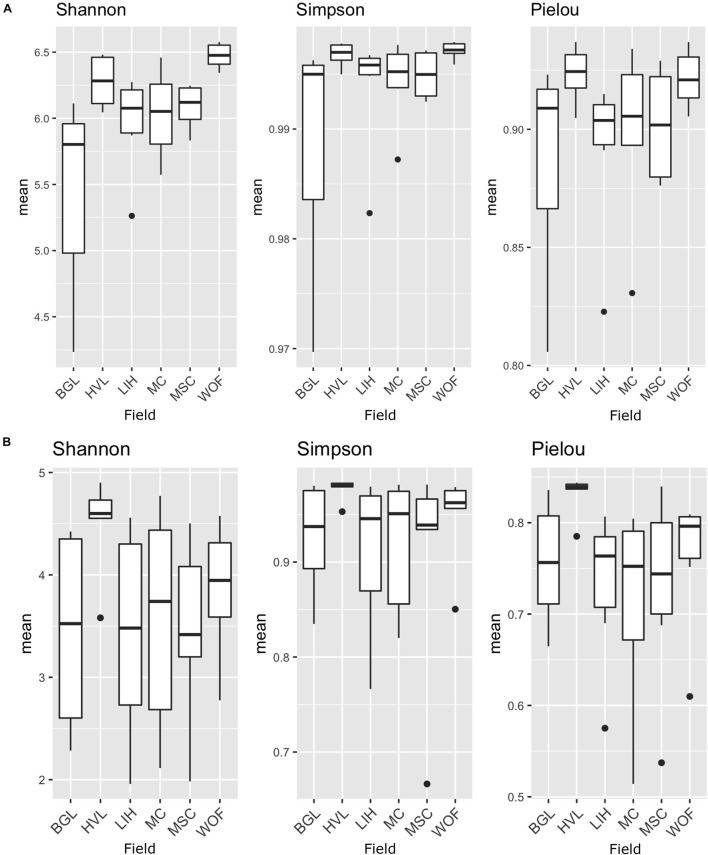
Microbial species diversity and evenness in different hemp fields. The analysis of alpha diversity indices (Shannon and Simpson) and Pielou’s evenness index of the bacterial and fungal communities: **(A)** bacterial microbiota; **(B)** fungal microbiota. Indices are shown according to fields. Field name: BGL, Bluegrass Lane; HVL, Hudson Valley Lab; LIH, Long Island Horticultural Research and Extension Center; MC, McCarthy CBD Trial; MSC, McCarthy Stress Trial; and WOF, Wegman’s Organic Farm. Three replicates were used for the analysis of species diversity and evenness.

**FIGURE 3:**
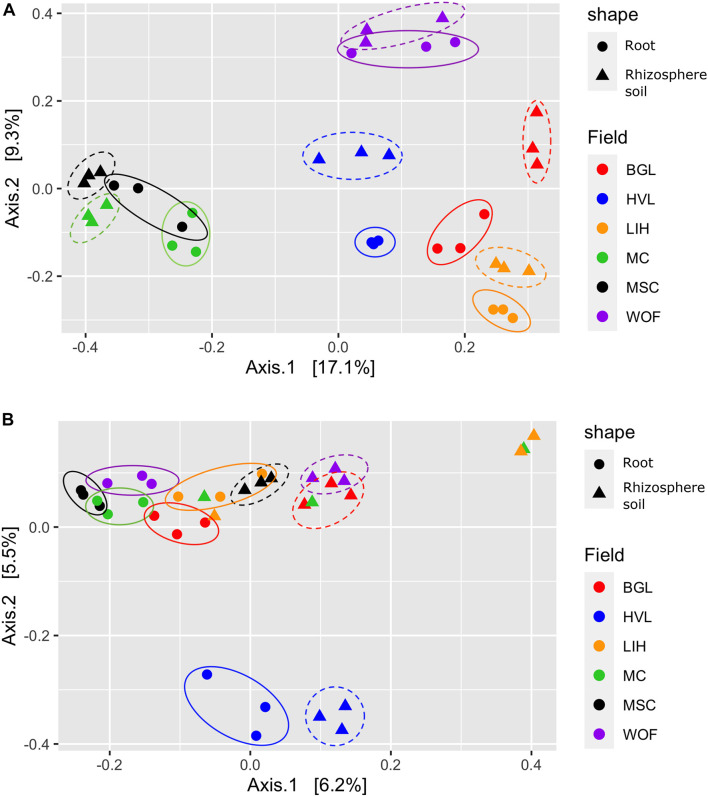
Structure of the community. Principal coordinates analysis (PCoA) showing the community compositions assignments of **(A)** bacterial 16S rDNA and **(B)** fungal ITS genes. The variance in the ordinations’ axes 1 and 2 is displayed in parenthesis. Samples from the rhizosphere soil and root are shown by circular and triangle shapes, respectively. Three replicates were used for PCoA analysis. Each color represents filed name: BGL, Bluegrass Lane; HVL, Hudson Valley Lab; LIH, Long Island Horticultural Research and Extension Center; MC, McCarthy CBD Trial; MSC, McCarthy Stress Trial; and WOF, Wegman’s Organic Farm.

The Shannon diversity index for bacteria is not as consistent as the Simpson index across all six hemp fields. The WOF field has the highest diversity mean in the Bacterial Shannon diversity index, whereas the BGL field has the lowest. However, the Simpson diversity index for all six fields is close to the maximum (Figure 2A). The Shannon diversity index was highly significant (*P* = 1.81E-05) on bacteria across fields (Supplementary Table 1). The BGL field was significant from HVL (*P* < 0.001), LIH (*P* = 0.02), MC (*P* = 0.008), MSC (*P* = 0.003), and WOF (*P* = 6.74E-06), and the LIH field was significant (*P* = 0.02) from WOF. The Pielou’s evenness index did not varied much but BGL field had the lowest mean evenness index and there is a significant difference (*P* = 0.04) of species evenness in the bacterial communities (Figure 2A and Supplementary Table 1). For Fungi, the HVL field has the highest mean diversity according to the Shannon diversity index and the difference was statistically significant (*P* = 0.01). The Simpson diversity index is quite homogenous across fields (Figure 2B) and there is no significant difference between hemp fields (Supplementary Table 1). Although the mean of Pielou’s evenness index is quite higher in HVL field, there is no statistically significant difference observed in the fungal communities across fields (Figure 2B). However, sample type (root or rhizosphere soil) had a significant effect on the bacterial (Shannon *P* = 1.12E-05, Simpson *P* = 0.001) and fungal diversity (Shannon *P* = 9.18E-08, Simpson *P* < 0.001). The Pielou’s evenness index also showed significant difference for sample type in the bacterial (Pielou *P* = 2.00E-05) and fungal communities (Pielou *P* < 001; Supplementary Table 1). The diversity of bacteria in the WOF plot, which was the only organic field in the study, tend to be high for Shannon and Simpson indices, but the difference was not highly noticeable. Organic and conventional fields have similar microbial diversity and community structure. Bacterial communities clustered by biotopes according to the PCoA ordination (Figures 3A,B). Root associated bacteria in HVL and LIH formed strong clusters out of six hemp fields. In the rhizosphere soil, the bacterial community structure was clustered in three fields: LIH, MC, and MSC (Figure 3A). That said, fungal communities clustered differently than bacterial communities. For root biotopes, only MSC displayed a clear clustering pattern, while rhizosphere soil biotopes were clustered strongly in three fields: HVL, MSC, and WOF (Figure 3B). Using the PERMANOVA test, we observed that hemp fields revealed highly significant variations in both compartments in the bacterial (root: *R*^2^ = 0.611, *P* = 0.001, and rhizosphere soil: *R*^2^ = 0.807, *P* = 0.001) and fungal (root: *R*^2^ = 0.425, *P* = 0.001, and rhizosphere soil: *R*^2^ = 0.485, *P* = 0.001) community structure (Table 2).

**TABLE 2:** Effect of fields on the structure of the bacterial and fungal communities in root and rhizosphere soil according to PERMANOVA.

Variable	Source	DF	SumOfSqs	*R* ^2^	*F*	Pr(>F)
**(A) Bacteria**						
Roots	Field	5	3.595	0.611	3.778	0.001***
	Residual	12	2.283	0.388		
	Total	17	5.878	1.000		
Rhizosphere soil	Field	5	3.676	0.807	10.069	0.001***
	Residual	12	0.876	0.192		
	Total	17	4.552	1.000		
**(B) Fungi**						
Roots	Field	5	2.946	0.425	1.774	0.001***
	Residual	12	3.985	0.575		
	Total	17	5.878	1.000		
Rhizosphere soil	Field	5	2.785	0.485	2.257	0.001***
	Residual	12	2.962	0.515		
	Total	17	5.748	1.000		

*** means significance at P = 0.001.

### Differential Taxonomic Profiles in Hemp-Associated Microbial Community

**FIGURE 4:**
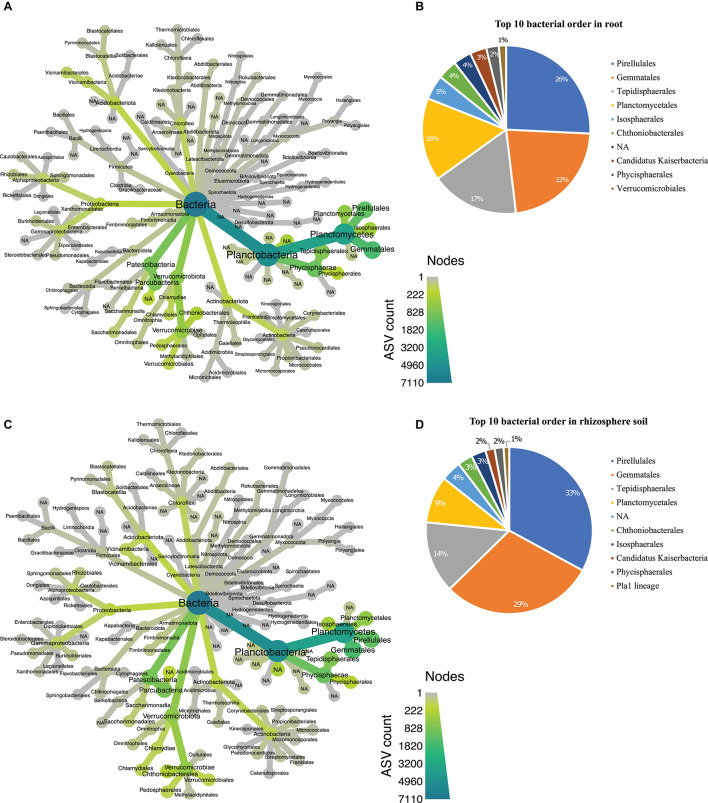
Taxonomic hierarchy abundance at the order level for bacterial communities. **(A)** Taxonomic representation at order level in root; **(B)** relative abundance of top 10 bacterial orders in root. **(C)** Taxonomic representation at order level in rhizosphere soil **(D)** relative abundance of top 10 bacterial orders in rhizosphere soil. Three replicates were used for this study.

In the bacterial communities, we found 26 abundant phyla (Supplementary Table 2), with Planctobacteria being the most abundant phyla in both root (Figure 4A) and rhizosphere soil (Figure 4C). The 7,134 bacterial ASVs (BASV) were assigned to 108 orders (Supplementary Table 3). We chose the top 10 orders based on their high relative abundance in both biotopes and eight of the 10 most abundant orders (Pirellulales, Gemmatales, Tepidisphaerales, Planctomycetales, Chthoniobacteriales, Isophaerales, Candidatus Kaiserbacteria, and Phycisphaerales) were dominant in both root and soil (Figures 4B,D). Both biotopes were dominated by Pirellulales (Figures 4B,D).

**FIGURE 5:**
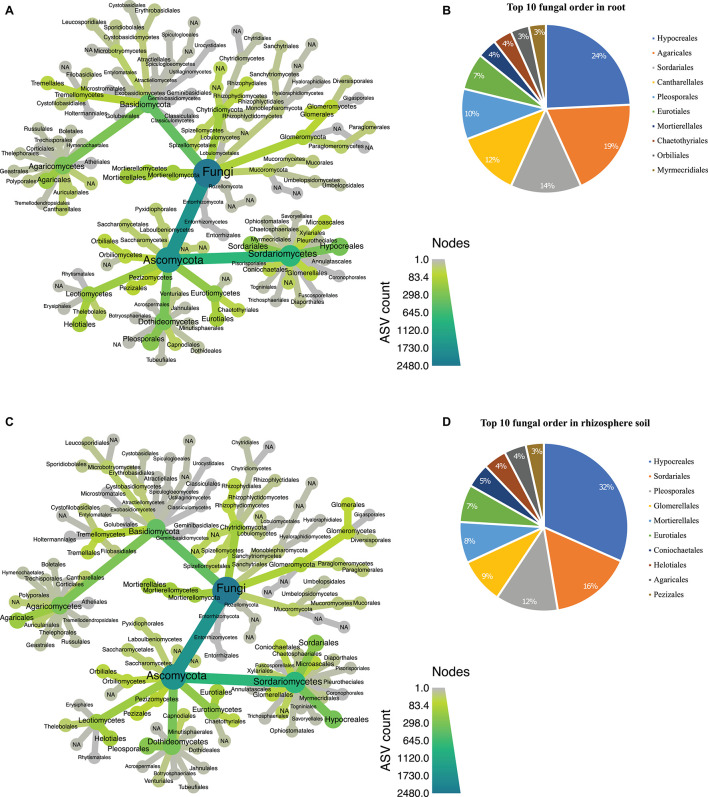
Taxonomic hierarchy abundance at the order level for fungal communities. **(A)** Taxonomic representation at order level in root; **(B)** relative abundance of top 10 fungal orders in root. **(C)** Taxonomic representation at order level in rhizosphere soil **(D)** relative abundance of top 10 fungal orders in rhizosphere soil. Three replicates were used for this analysis.

In the fungal dataset, we identified 12 phyla (Supplementary Table 4), with Ascomycota being the most abundant in both root (Figure 5A) and rhizosphere soil (Figure 5C). The 3,132 fungal ASVs were classified into 110 orders (Supplementary Table 5). The order Hypocreales dominated both biotopes followed by Agaricales, Sordariales, Cantharellales, Pleosporales, Eurotiales, Mortierellales, Chaetothyriales, Orbiliales, and Myrmecridiales (Figures 5B,D). Based on relative abundance, Hypocreales, Sordariales, Pleosporales, Glomererellales, Mortierellales, Eurotiales, Coniochaetales, Heliotales, Agaricales, and Pezizales were the top 10 fungal orders in the rhizosphere soil (Figure 5D). Both the root and rhizosphere soil fungal communities shared six (Hypocreales, Agaricales, Sordariales, Pleosporales, Eurotiales, and Mortierellales) of the top 10 orders (Figures 5B,D).

### Indicator Species and Core Microbiota Across Six Hemp Fields

**FIGURE 6:**
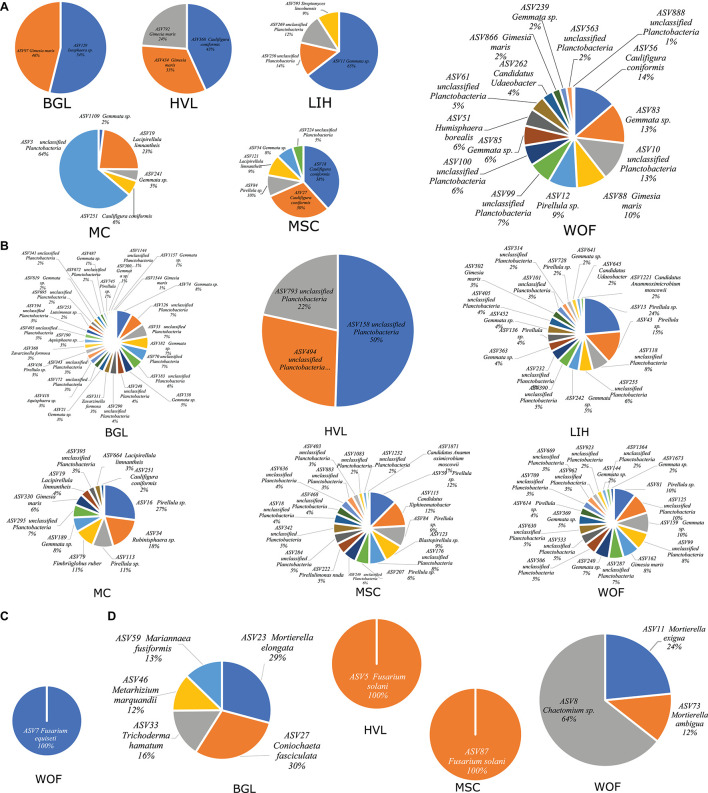
Indicator species of hemp microbiome. The most representative species of hemp microbiome were identified through indicator species analysis in six different field locations. Bacterial indicator species in root **(A)** and in rhizosphere soil **(B)**; and fungal indicator species in root **(C)**; and in rhizosphere soil **(D)**. Fungal indicator species in root identified only in WOF filed. Three replicates were used for analyzing indicator species in each field.

The indicator species analysis with Šidák correction for multiple comparisons accounted for on average 1 to 65% relative abundance of all root bacteria and 1 to 50% relative abundance of all rhizosphere soil bacteria detected in six different fields (Figure 6). However, fungal indicator species were not found in every field, for example, ASV7 (*Fusarium equiseti*) found in root in WOF (Figure 6C), in HVL and MSC one ASV was identified in the rhizosphere soil (Figure 6D). In root, WOF had the greatest number of bacterial indicator species (15), while in the rhizosphere soil, BGL had the most (30; Figure 6B). BGL had a maximum of six fungi in the rhizosphere soil (Figure 6D and Supplementary Table 6). Core microbiome composed of a small number of relatively abundant bacterial and fungal ASVs for each field. Here, we used the term “BASV” defining Bacterial ASV and “FASV” for fungal ASV. We found eight BASV as rhizosphere soil core microbiota, which were identified as four Planctobacteria (*Gimesia maris, Pirellula sp. Lacipirellula limnantheis*, and *Gemmata sp.*) and three of which were Unclassified Planctobacteria (Supplementary Figure 1A and Supplementary Table 7). *Fusarium oxysporum*, *Gibellulopsis piscis*, and *Fusarium equiseti* were identified as fungal core microbiota (Supplementary Figure 1B and Supplementary Table 8).

### Network Patterns of Microbial Community in Field Grown Hemp

**FIGURE 7:**
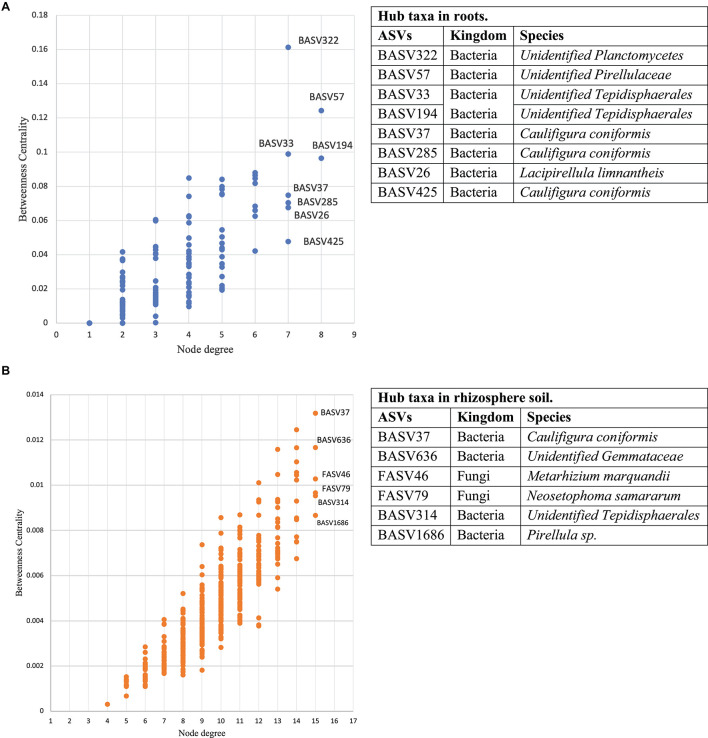
Interkingdom network cooccurrence in hemp microbiome. The microbial network cooccurrence includes three fully independent replicates. Betweenness centrality and node degree of ASVs and their inter-connection identified eight hub taxa in the root **(A)**. Betweenness centrality and node degree of ASVs and their inter-connection identified six hub taxa in rhizosphere soil **(B)**. Here soil refers to rhizosphere soil. Tables next to each figure identifies microbiota corresponding to the hub ASVs.

**FIGURE 8:**
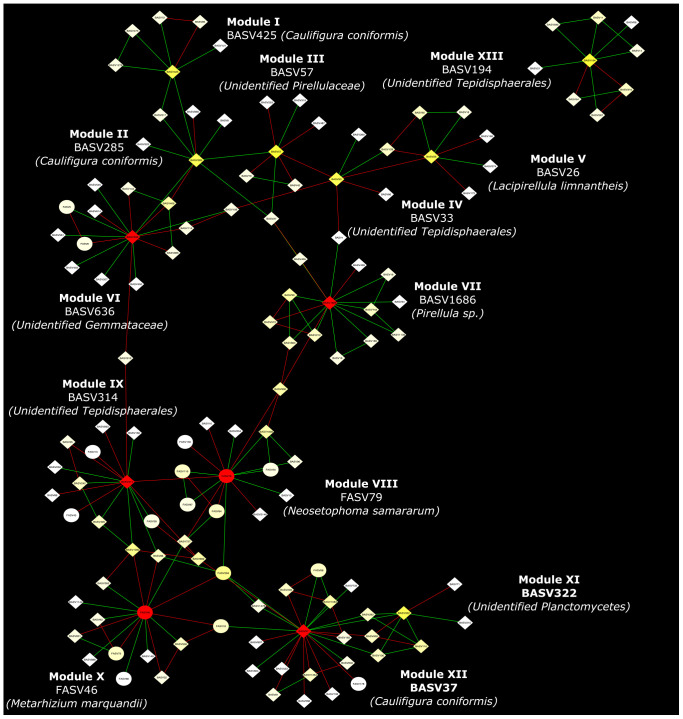
Network hub taxa identified across six hemp fields in New York. Interkingdom interactions of hub taxa in the root and rhizosphere soil was analyzed using three replicates. The co-association of each hub taxa and its co-associated ASVs is denoted as a Module and ASV connecting between Modules considered as connector taxa. Hub taxa of the module and connector taxa combinedly considered as Network hub taxa. Here soil refers to rhizosphere soil.

Co-occurrence network analysis of the hemp microbiota revealed complex co-occurrences among bacterial and fungal ASVs in the soil than in the root, of which 167 nodes and 257 edges in the root and 448 nodes and 2,164 edges in the rhizosphere soil. Based on the betweenness centrality score and node degree, eight BASV, BASV26 (*Lacipirellula limnantheis*), BASV33 (Unidentified Tepidisphaerales), BASV37 (*Caulifigura coniformis*), BASV57 (Unidentified Pirellulaceaes), BASV194 (Unidentified Tepidisphaerales), BASV285 (*C. coniformis*), BASV322 (Unidentified Planctomycetes), and BASV425 (*C. coniformis*) were identified as hub taxa in the root (Figure 7A and Supplementary Figure 2A). Four BASV, BASV37 (*C. coniformis*), BASV314 (Unclassified Tepidisphaerales), BASV636 (Unclassified Gemmatales), BASV1686 (*Pirellula sp.*), and two fungal ASVs, FASV46 (*Metarhizium marquandii*) and FASV79 (*Nectria ramulariae*), were identified as hub microbes in the rhizosphere soil microbiome network (Figure 7B and Supplementary Figure 2B). BASV37 (*C. coniformis*) was the only hub taxa found both in the root and rhizosphere soil (Figure 7 and Supplementary Figure 2). The network of each hub taxon and its connected ASVs is termed to as a module, and Modular hub taxa were ASV-centered within a module. We identified a meta co-occurrence pattern of 13 modules (Figure 8). We also identified taxa connecting different modules and considered as connector taxa, and the taxa connecting within modules and in general are considered as network hub taxa. Detailed taxonomic information of modular hub, connector and network hub can be found in the Table 3. Their co-occurrence pattern revealed: (i) root modular hub ASVs, BASV425, BASV285, BASV57, BASV33, and BASV26 are connected, and four connector ASVs, BASV406, BASV108, BASV27, and BASV1 established co-occurrences between rhizosphere soil modular hub (BASV636 and BASV1686) and root modular hub taxa; (ii) rhizosphere soil modular hub BASV636 and BASV314 are negatively co-occurred via BASV916; (iii) rhizosphere soil hub taxa FASV79 had negative co-occurrences with BASV314 and BASV1686; (iv) a connector taxon, BASV1090 had positive co-occurrence with rhizosphere soil modular hub BASV314 but negative co-occurrence with the modular hub FASV46; (v) FASV264 connected maximum four modular hub taxa (FASV46, FASV79, BASV37, and BASV314). BASV27 positively co-occurrence two rhizosphere soil modular hubs, BASV285 and BASV57, but negatively with a rhizosphere soil modular hub taxon, BASV1686. BASV57 established positive co-occurrences with a rhizosphere soil modular hub FASV46 and negative co-occurrences with two rhizosphere soil modular hubs, BASV314 and FASV79; (vi) FASV18 was positively connected with BASV37 but negatively with FASV46; and (vii) root modular hub BASV194 was not found in the co-occurrences network of network hub taxa. We identified eight bacterial and two fungal ASVs connecting all modular hub taxa to extend networks between the root and rhizosphere soil microbiota. Thus, these 10 connector ASVs and modular hubs, for a total of 24 ASVs have been chosen as network hub taxa (Figure 8 and Table 3). These 23 network hub taxa were identified as four bacteria and three fungi, and five ASVs were unidentified at the genus level were considered as network hub microbiota in the hemp microbiome network across six different field sites (Table 3).

**TABLE 3:** Network hub taxa identified in six hemp fields.

ASV_ID	Kingdom	Phylum	Class	Order	Family	Genus	Species
BASV57	Bacteria	Planctobacteria	Planctomycetes	Pirellulales	Pirellulaceae	*Unidentified Pirellulaceae*	*Unidentified Pirellulaceae*
BASV194	Bacteria	Planctobacteria	Phycisphaerae	Tepidisphaerales	WD2101 soil group	*Unidentified Tepidisphaerales*	*Unidentified Tepidisphaerales*
BASV322	Bacteria	Planctobacteria	Planctomycetes	Planctomycetales	Rubinisphaeraceae	*Unidentified Planctomycetes*	*Unidentified Planctomycetes*
BASV33	Bacteria	Planctobacteria	Phycisphaerae	Tepidisphaerales	WD2101 soil group	*Unidentified Tepidisphaerales*	*Unidentified Tepidisphaerales*
BASV37	Bacteria	Planctobacteria	Planctomycetes	Planctomycetales	Rubinisphaeraceae	*Caulifigura*	*Caulifigura coniformis*
BASV285	Bacteria	Planctobacteria	Planctomycetes	Planctomycetales	Rubinisphaeraceae	*Caulifigura*	*Caulifigura coniformis*
BASV26	Bacteria	Planctobacteria	Planctomycetes	Pirellulales	Lacipirellulaceae	*Lacipirellula*	*Lacipirellula limnantheis*
BASV425	Bacteria	Planctobacteria	Planctomycetes	Planctomycetales	Rubinisphaeraceae	*Caulifigura*	*Caulifigura coniformis*
BASV636	Bacteria	Planctobacteria	Planctomycetes	Gemmatales	Gemmataceae	*Unidentified Gemmataceae*	*Unidentified Gemmataceae*
FASV46	Fungi	Ascomycota	Sordariomycetes	Hypocreales	Clavicipitaceae	*Metarhizium*	*Metarhizium marquandii*
FASV79	Fungi	Ascomycota	Dothideomycetes	Pleosporales	Phaeosphaeriaceae	*Neosetophoma*	*Neosetophoma samararum*
BASV314	Bacteria	Planctobacteria	Phycisphaerae	Tepidisphaerales	WD2101 soil group	*Unidentified Tepidisphaerales*	*Unidentified Tepidisphaerales*
BASV1686	Bacteria	Planctobacteria	Planctomycetes	Pirellulales	Pirellulaceae	*Pirellula*	*Pirellula sp.*
BASV406	Bacteria	Planctobacteria	Planctomycetes	Gemmatales	Gemmataceae	*Gemmata*	*Gemmata sp.*
BASV108	Bacteria	Planctobacteria	Planctomycetes	Pirellulales	Pirellulaceae	*unidentified Pirellulaceae*	*unidentified Pirellulaceae*
BASV1	Bacteria	Planctobacteria	Planctomycetes	Pirellulales	Lacipirellulaceae	*Lacipirellula*	*Lacipirellula limnantheis*
BASV916	Bacteria	Planctobacteria	Planctomycetes	Gemmatales	Gemmataceae	*Unidentified Gemmataceae*	*Unidentified Gemmataceae*
BASV960	Bacteria	Planctobacteria	Planctomycetes	Pirellulales	Pirellulaceae	*Pirellula*	*Pirellula sp.*
BASV1090	Bacteria	Planctobacteria	Phycisphaerae	Pla1 lineage	NA	*Unidentified Phycisphaerae*	*unidentified Phycisphaerae*
BASV27	Bacteria	Planctobacteria	Planctomycetes	Planctomycetales	Rubinisphaeraceae	*Caulifigura*	*Caulifigura coniformis*
BASV757	Bacteria	Planctobacteria	Planctomycetes	Gemmatales	Gemmataceae	*Unidentified Gemmataceae*	*Unidentified Gemmataceae*
FASV264	Fungi	Ascomycota	Sordariomycetes	Hypocreales	Clavicipitaceae	*Metarhizium*	*Metarhizium marquandii*
FASV18	Fungi	Ascomycota	Sordariomycetes	Hypocreales	Nectriaceae	*Fusarium*	*Fusarium oxysporum*

## Discussion

We studied the microbial communities associated with a single hemp cultivar growing in six field locations in New York State and different agricultural practices. Although the number of samples per field was low which may be a factor that influence statistical analyses, we therefore focus on comparisons of microbial communities in biotopes, the core, and the network across all samples. In hemp root and soil, we showed the diversity of bacterial and fungal communities, taxonomic abundance and interkingdom interactions. Planctobacteria and Ascomycota dominated bacterial and fungal communities in hemp microbiota in six fields. Eight BASV and three fungal ASVs dominated the core microbiota. We discovered 24 ASVs as network hub taxa that are establishing microbial network associated with *C. sativa* TJ’s CBD.

The alpha diversity of the microbial communities was greatly affected by compartment, but we found that community structure varied significantly among different hemp field sites. The community structure observed in this study resembled patterns found previously in hemp (*C. sativa* “Anka”) growing in six fields in New York’s Finger Lakes region (Barnett et al., 2020). Some of the main contributors to the taxa abundance in hemp in this study was Planctobacteria which was highly enriched in root and rhizosphere soil biotopes, showed similar trends in the root and rhizosphere soil in other crops, such as soybean (Ahmed et al., 2021), soybean straw returns (Miao et al., 2020), and wild beet (Zachow et al., 2014). Planctomycetes (Planctobacteria) were observed in the highly abundant (>1% of the community) bacteria in multiple compartment (root, rhizosphere soil, bulk soil, flowers, and leaves) in field-grown hemp (Barnett et al., 2020). In support of Barnett et al. (2020) report of hemp associated fungi, we identified four of the top 10 fungal orders that were proven to have a greater association of those microbes with hemp, providing evidence that those fungal ASVs has an important impact in field-grown hemp in New York. For root and rhizosphere soil communities, the influence of field site was strongest, implying that several factors including soil chemistry were likely to be driving this field specific variance. Our study comprised six different field locations, each with unique soil features that could influence hemp-associated microbiota. Overall, our data indicated that while local environment factors may influence hemp microbiota, certain bacteria and fungi were abundant in hemp irrespective of fields location.

To learn more about microbial community patterns across fields and hemp-microbe interactions, we identified indicator species and the core microbiome. The indicator species analysis identifying different bacteria and fungi and their ties to field sites allows in predicting their presence and abundance of those taxa. In comparing to other fields, the rhizosphere fungal communities in LIH and MC are scattered. In LIH and MC, we found no fungal indicator species in the root and rhizosphere soil, which could explain why the LIH and MC fungal communities in our PCoA were so dispersed (Figure 3B). We found *Streptomyces lincolnensis* as an indicator species associated with hemp roots in LIH field. However, *Streptomyces sp.* has been reported as a potential biocontrol agent of plant pathogens as well as of plant growth enhancer (Figueiredo et al., 2010; de Jesus Sousa and Olivares, 2016; Vurukonda et al., 2018). Beneficial fungal species, *Trichoderma hamatum* and *Metarhizium marquandii*, were identified as fungal indicator species in the rhizosphere soil of BGL field. It has been proven that *T. hamatum* has biocontrol activity and potential to increase plant biomass extensively in agriculture practices (Harman et al., 2004; Studholme et al., 2013), such as it promotes lettuce growth in low pH and nutrient poor soil, and protect against pre-emergence disease caused by *Rhizoctonia solani* and *Sclerotinia sclerotiorum* (Ryder et al., 2012). Recently, *Metarhizium marquandii* was selected from an *in vitro* screening with another fungal species and observed in solubilizing phosphorus, producing indoleacetic acid and contributing to plant growth in soybean, maize, and bean (Baron et al., 2020).

No core microbiota was identified in hemp root, and a major portion of the bacterial core (37%) was unclassified at the genus level, indicating that the core hemp microbiota comprises bacteria that have yet to be isolated or documented. This resulted in a list of microbes that should now be prioritized for directed isolation or genome binning using sequencing to further understand their potentials in hemp. We observed very little evidence of core microbiota. We identified a limited list of microbes as core microbiota (four bacteria and three fungi), which is similar to recent field-grown hemp study, who identified five bacterial and fungal core taxa in the bulk soil (Barnett et al., 2020). *Fusarium solani* identified as soil indicator species in HVL and MSC field sites. *Fusarium oxysporium* has been identified as a core fungal microbiota. *Fusarium* are documented for its devastating effects such as causing root and crow rot of *C. sativa* (Punja, 2021). But the presence of *Fusarium* as a core microbiota in bulk soil of field-grown hemp (Barnett et al., 2020), not only supports its common occurrence but also opens the possibility of studying the interactions of host-dependent pathogens to truly comprehend soil health of hemp fields. *Fusarium* spp. could be combined with other biocontrol agents to enhance hemp protection against pathogen attacks (Kaur et al., 2011). Beneficial bacteria may colonize *Fusarium* species, which could explain the positive correlations on the one hand and the hemp benefits on the other, which have been observed in canola (Lay et al., 2018), non-pathogenic *F. oxysporum* isolate Fo47 discovered from *Fusarium* wilt suppressive soils in France, has been intensively studied for the management of *Fusarium* wilt disease in a variety of vegetable and flower crops (Alabouvette et al., 1998). However, we did not investigate *Fusarium*’s non-pathogenic activity in hemp. Therefore, future studies on functional activities, including biocontrol agents is needed. Our findings illustrate a list of prospective candidate microbes for further research into hemp-specific interactions and how their activity relates to hemp performance.

Plant microbial networks are complicated, and each contributing taxon performs specialized roles that are critical to the ecosystem’s functionality, as a result, contribute to plant health (Zhou et al., 2010; Huang et al., 2020). Thus, it is essential to study microbial co-occurrences in the plant microbiome and how they impact plant health. We identified 24 ASVs as network hub taxa that maintain microbial community structure in root and rhizosphere soil. Since the hub microbes are crucial to plant health (Philippot et al., 2013; Agler et al., 2016), they could be ideal targets for new management practices intended to boost crop production. Microbial hub taxa can also link factors controlling the dynamics of plant associated microbiome and more effectively stabilizing selected microbiota according to plant genotype (Agler et al., 2016). As a result, their identity as well as functional role should be clarified. Addressing the microbial co-occurrences with hemp can enhance crop health and productivity by manipulating grow strategies. Given the fact that study into the hemp microbiome is still in its preliminary phase, data suggest that cultivar and soil type dependent selection are the primary factors in determining microbial community composition (Garbeva et al., 2004; Berg and Smalla, 2009; Taghinasab and Jabaji, 2020). We detected *Chaetomium globosum* as an indicator species in the rhizosphere soil of the WOF field (Supplementary Table 6), which confirms the previous reports of hemp endophytes (Kusari et al., 2013). Most of the microbiome studies in *C. sativa* have focused on high THC cultivars (McKernan et al., 2016; Comeau et al., 2020) as well as identifying endophytes (Gautam et al., 2013; Kusari et al., 2013; Scott et al., 2018; Taghinasab and Jabaji, 2020). However, there has been little progress in understanding of endophytic bacteria and fungi in various organs affects growth and biocontrol potentiality (Pagnani et al., 2018; Taghinasab and Jabaji, 2020) but the underlying mechanism is still unexplained. It’s been proposed that bacteria isolated from one plant species might improve plant biomass and respond to stress in another, implying that beneficial microbes could enhance hemp production. Our results suggest that searching for field sites reliant microbial community assemblages can enhance hemp biomass, biocontrol pathogens, and secondary metabolites production, as previously explained (Taghinasab and Jabaji, 2020). The discovery of core and hub microbial members in field-grown hemp in New York state not only verifies the importance of common microbes in screening and assemblage of hemp microbiota (Barnett et al., 2020), but also opens up new prospects in studying whether there are any hemp-associated bacteria or fungus that could be used to produce biocontrol agents.

Plant microbiomes have huge potential to increase yield in a sustainable way to meet rising global food and biofuel demands. Substantial investigations are necessary to correctly assess the roles of microbial interactions with hemp in modern agricultural systems with the aim of integrating beneficial microbiomes into crop yields. By studying microbial diversity, community structure and interkingdom interactions between bacteria and fungi in hemp roots and rhizosphere soil in natural fields, as well as defining core and hub microbiota for further inquiry and manipulations, our study contributed to addressing key information gaps for hemp cultivar-by-environment-microbiota interactions. Understanding the role of microbiome in distinct plant compartments and different developmental stages, as well as their role on the quality and quantity of yield and secondary metabolites production has been given importance (Barnett et al., 2020; Dastogeer et al., 2020; Taghinasab and Jabaji, 2020). Our study based on the microbiota at maturity stage; nevertheless, we may have missed some microbial members during other developmental stages. However, our findings endorse the idea that some bacterial and fungal taxa were shared by the clonal hemp cultivar “TJ’s CBD” grown across six different field locations, and that such taxa seemed to be strongly associated with hemp regardless of location. This study could be effective in adding more understanding into the role of hemp-associated bacteria and fungus throughout plant development by merging microbiome and transcriptomics data.

## Data Availability Statement

The datasets presented in this study can be found in online repositories. The names of the repository/repositories and accession number(s) can be found below: all sequences are accessible in NCBI SRA database under the accession number PRJNA740043.

## Author Contributions

BA: conceptualization, experimental design, processed data, and wrote the manuscript. LS: conceptualization, experimental design, sample collection, supervision, and manuscript editing. MH: concepts, design, supervision, and contribution to the manuscript writing. All authors contributed to the article and approved the submitted version.

## Conflict of Interest

The authors declare that the research was conducted in the absence of any commercial or financial relationships that could be construed as a potential conflict of interest.

## Publisher’s Note

All claims expressed in this article are solely those of the authors and do not necessarily represent those of their affiliated organizations, or those of the publisher, the editors and the reviewers. Any product that may be evaluated in this article, or claim that may be made by its manufacturer, is not guaranteed or endorsed by the publisher.

## Footnotes

1
http://bioinformatics.psb.ugent.be/webtools/Venn/
