# Supplementary material for: Microbiome of Field Grown Hemp Reveals Potential Microbial Interactions With Root and Rhizosphere Soil
Source: Front Microbiol. 2021 Nov 15;12:741597. doi: 10.3389/fmicb.2021.741597 (PMC8634612; doi:10.3389/fmicb.2021.741597)
Supplement: Supplementary file 1 [file Data_Sheet_1.docx]

**Supporting Information**

**Title: Microbiome of field grown hemp reveals potential microbial interactions with root and rhizosphere soil**

**Bulbul Ahmed, Lawrence B. Smart and Mohamed Hijri**

This article has the following supporting information:

**Methods S1. Sequence processing and analysis of data**

The microbiome pipeline DADA2 has been used to process, align, and analyse MiSeq reads to get distinct sequence variants known as Amplicon Sequence Variants (ASV) (Callahan, et al., 2016). Using the filterAndTrim function (minLen = 50, maxN=0, maxEE=c(3,3), trancQ=2) (details in the complementary R scripts), reads were trimmed to achieve comprehensive quality thresholds by deleting primers and low-quality sequences, followed by filtering with DADA2's error model process using the learnErrors function. After that, the ASV table was created by deduplicating, sample interference, merging pair-end readings, and removing chimaera. The reference database was used to assign taxonomy using the function assignTaxonomy using SILVA for 16S rRNA (Quast, et al., 2013), and UNITE database for ITS (Nilsson, et al., 2018). Non-fungal ASVs were removed from the fungal taxonomy dataset. BLASTn on NCBI was used to manually test the identity of the ASVs of interest (Johnson, et al., 2008). The bioinformatics workflow was run remotely on an arcade's multithread computer on arcade (<http://www.esi.umontreal.ca/connexion_ssh.html>). We used the rarecurve function of the vegan package (Oksanen, et al., 2019) to normalise the dataset to the lowest number of reads assembled for each sample by selecting randomly subsampling of the read data from each sample. The relative abundance of taxa in each family was analyzed using the package dplyr v2.0.0 (Wickham and Wickham, 2020) in R4.0.2 (Team, 2020). We deleted ASVs taxonomically attributed to chloroplast and mitochondria from both the bacterial and fungal databases, assuming they were likely to be part of the plant genome. The Shannon and Simpson’s Alpha diversity indices were calculated in R using the vegan package v.2.5-6. Analysis of variance (ANOVA) was used to determine the impact of treatments on diversity indices and Tukey’s *post-hoc* tests was used to compare treatments and sample types using the R package agricolae v1.3-3 (Peșteanu and Bostan, 2020). Using the R package vegan v 2.5.6 (Oksanen, et al., 2020), the structure of the bacterial and fungal communities (Beta diversity) was analysed using Principal Coordinates Analysis (PCoA) based on Bray-Curtis distances. To see whether the different treatments and sample types have an impact on community composition as a constant variable, we performed PERmutational Multivariate ANalysis Of VAriance (PERMANOVA) (Anderson, 2001) with the function Adonis of the R package vegan v 2.5-6. To test significance, the abundance matrix of ASV was Hellinger-transformed and 999 permutations were utilised (Oksanen, et al., 2019). Using the R package metacoder v 0.3.4, we displayed taxonomic abundance at the order level of community composition (Poisot, et al., 2017). We used the software indicspecies v 1.7.9 to analyse indicator species (De Cáceres and Jansen, 2019) in R4.0.2. using Šidák correction for multiple comparison in the R package ‘RVAideMemoire’ v 0.9-78 (Hervé and Hervé, 2020). The ASVs found in all plots' microbial assemblages are called the core microbiota. Using the method glasso from the package SPIEC-EASI v 1.0.6 (Kurtz, et al., 2015), we built a co-occurrence network to examine the relationships between ASVs from the root and rhizosphere microbiomes. The networks were then plotted in Cytoscape v 3.8.0 (Shannon, et al., 2003). In multiparticle interactions, betweenness centrality and degree of connectivity scores of >95 percent of the taxa in the network were considered, allowing the highly linked taxa to be labelled as hub taxa.

**References**

Anderson, M.J. (2001) A new method for non‐parametric multivariate analysis of variance, *Austral ecology* **26**: 32-46.

Callahan, B.J., McMurdie, P.J., Rosen, M.J., Han, A.W., Johnson, A.J., and Holmes, S.P. (2016) DADA2: High-resolution sample inference from Illumina amplicon data, *Nat Methods* **13**: 581-583.

De Cáceres, M., and Jansen, F. (2019) indicspecies-package: Studying the statistical relationship between species and.

Hervé, M., and Hervé, M.M. (2020) Package ‘RVAideMemoire’, *See* [*https://CRAN*](https://CRAN)*. R-project. org/package= RVAideMemoire*.

Johnson, M., Zaretskaya, I., Raytselis, Y., Merezhuk, Y., McGinnis, S., and Madden, T.L. (2008) NCBI BLAST: a better web interface, *Nucleic Acids Res* **36**: W5-9.

Kurtz, Z.D., Muller, C.L., Miraldi, E.R., Littman, D.R., Blaser, M.J., and Bonneau, R.A. (2015) Sparse and compositionally robust inference of microbial ecological networks, *PLoS Comput Biol* **11**: e1004226.

Nilsson, R.H., Larsson, K.-H., Taylor, A.F S., Bengtsson-Palme, J., Jeppesen, T.S., Schigel, D., et al. (2018) The UNITE database for molecular identification of fungi: handling dark taxa and parallel taxonomic classifications, *Nucleic Acids Research* **47**: D259-D264.

Oksanen, J., Blanchet, F., Friendly, M., Kindt, R., Legendre, P., McGlinn, D., et al. (2019) vegan: Community Ecology Package. R package version 2.5-6. 2019.

Oksanen, J., Blanchet, F., Friendly, M., Kindt, R., Legendre, P., Mcglinn, D., and Stevens, M. (2020) Package—vegan: Community ecology package. R package version 2.5-6.

Peșteanu, A., and Bostan, M. (2020) Perfecţionarea unor elemente tehnologice la producerea materialului săditor pentru fondarea livezilor moderne de măr, *Stiinta agricola*: 52-59.

Poisot, T., Foster, Z.S.L., Sharpton, T.J., and Grünwald, N.J. (2017) Metacoder: An R package for visualization and manipulation of community taxonomic diversity data, *PLOS Computational Biology* **13**.

Quast, C., Pruesse, E., Yilmaz, P., Gerken, J., Schweer, T., Yarza, P., et al. (2013) The SILVA ribosomal RNA gene database project: improved data processing and web-based tools, *Nucleic Acids Res* **41**: D590-596.

Shannon, P., Markiel, A., Ozier, O., Baliga, N.S., Wang, J.T., Ramage, D., et al. (2003) Cytoscape: a software environment for integrated models of biomolecular interaction networks, *Genome Res* **13**: 2498-2504.

Team, R.C. (2020) R: A Language and Environment for Statistical Computing. Vienna: R Project.

Wickham, H., and Wickham, M.H. (2020) Package ‘plyr’, *Obtenido de* [*https://cran*](https://cran)*. rproject. org/web/packages/dplyr/dplyr. pdf*.

**Table S1.** The effects of fields and type of samples (root or rhizosphere soil) to hemp on alpha diversity and community structure of bacteria and fungi.

|  | **Bacteria** | | | | | | **Fungi** | | | | | |
| --- | --- | --- | --- | --- | --- | --- | --- | --- | --- | --- | --- | --- |
|  | **Shannon** | | **Simpson** | | **Pielou’s Evenness** | | **Shannon** | | **Simpson** | | **Pielou’s Evenness** | |
|  | ***F*** | ***Pr(>F)*** | ***F*** | ***Pr(>F)*** | ***F*** | ***Pr(>F)*** | ***F*** | ***Pr(>F)*** | ***F*** | ***Pr(>F)*** | ***F*** | ***Pr(>F)*** |
| **Field** | 10.614 | 1.81E-05 | 2.952 | 0.032 | 2.776 | 0.040 | 3.832 | 0.01 | 1.218 | 0.330 | 2.258 | 0.081 |
| **Sample type** | 30.477 | 1.12E-05 | 13.739 | 0.001 | 27.968 | 2.00E-05 | 56.640 | 9.14E-08 | 17.743 | <0.001 | 17.487 | <0.001 |
| **Field: Sample type** | 2.715 | 0.044 | 1.779 | 0.155 | 0.854 | 0.525 | 1.390 | 0.276 | 0.842 | 0.533 | 0.734 | 0.604 |

**Figure S1. Core microbiota in hemp microbiome.** Fungal core microbiota in rhizosphere soil (A) and bacterial core microbiota in rhizosphere soil (B). Here soil refers to rhizosphere soil.

**Figure S2. Hub taxa identified in the interkingdom network.** (A) network of hub taxa in the root; (B) network of hub taxa in the rhizosphere soil compartment. Here soil refers to rhizosphere soil.

**Supplementary Tables**

**Supplementary Tables S1-S7 are shown in Excel File.**
